# Diagnostic Accuracy of Ultrasonography in Diagnosis of Metatarsal Bone Fracture; a Cross Sectional Study

**Published:** 2019-08-17

**Authors:** Mohsen Ebrahimi, Seyed Reza Habibzadeh, Sayyed Reza Ahmadi, Samaneh Khajeh Nasiri, Mohammad Majid Kaveh, Mahdi Foroughian

**Affiliations:** 1 Department of Emergency Medicine, Faculty of Medicine, Mashhad University of Medical sciences, Mashhad, Iran.; 2 Department of Radiology, Faculty of Medicine, Mashhad University of Medical sciences, Mashhad, Iran.

**Keywords:** Ultrasonographyradiography fractures, bone, metatarsal bones

## Abstract

**Introduction:**

Metatarsus is one of the most common sites in the sole of foot bones fractures. The aim of this study was to determine the diagnostic accuracy of ultrasound in diagnosis of metatarsal bone fractures following foot trauma.

**Methods:**

This cross-sectional study was carried out on patients with blunt foot trauma admitted to emergency department of a hospital in Mashhad, Iran from January to September 2016. All patients were evaluated with bedside ultrasound for the presence of first to fifth metatarsal fractures and screening performance characteristics of ultrasonography in detection of metatarsal fractures were calculated considering foot radiography as the reference test.

**Results:**

The study was conducted on 102 patients with a mean age of 35.14±14.32 years (56.8% male). The most common signs of trauma in physical examination were pain and tenderness (100%), swelling (96.1%), ecchymosis (14.7%) and deformity (1.9%). Sensitivity, specificity, and positive and negative likelihood ratio of ultrasonography in detection of metatarsal bone fracture were 96.7% (95% CI: 0.83-0.99), 84.5% (95% CI: 0.73-0.92), 73.1% (95% CI: 0.57-0.85), and 98.3% (95% CI: 0.91-0.99), respectively. The overall accuracy of ultrasonography was 0.906 (95% CI: 0.844 – 0.969) based on area under the receiver operating characteristic (ROC) curve.

**Conclusion:**

Considering the excellent diagnostic accuracy, ultrasonography can be used as an alternative means in diagnosis of metatarsal bone fractures.

## Introduction

Foot and ankle are areas of the body that are most commonly exposed to trauma (1, 2). Foot injuries account for about 25% of musculoskeletal injuries leading to emergency department visits (3). Although such injuries usually are not considered life threatening, but the function of involved organs could be at risk. Therefore, correct and rapid diagnosis of these injuries as well as early treatment can prevent long-term and short-term complications (4, 5). On the other hand, it should be noted that accurate diagnosis of injuries to the foot bones, particularly metatarsal injuries, is not possible with physical examination alone and imaging procedures always have got a foothold, at least to rule out metatarsal fractures, as one of the most important clinical decisions (6, 7). Over time, the use of ultrasound (US) imaging has grown due to its low cost and lack of harmful radiation and good capability to check the soft tissue and its importance in diagnosis of foot and ankle injuries has increased (8). Some studies have evaluated the use of ultrasound in emergency departments for assessing the existence of foreign bodies (9) as well as examining the skeletal structures in many rheumatic diseases (10). Since US is considered as a method of dynamic measurements, it accurately detects the smallest changes in the anatomical structure of body and is very useful in patients with no specific clinical symptoms (3, 6, 11). To date, foot and ankle trauma is one of the most common indications in ultrasound assessment. In addition, patients usually cooperate for ultrasound (8, 12). On the other hand, because the same physician who examined the patient performs the ultrasound, it also provides more rapid diagnosis; since the doctor can easily match up findings of history taking with imaging and reach the best diagnosis.

In studies that have been conducted recently, the accuracy of the US in diagnosis of foot and ankle injuries has been reported as very good and the results have increased willingness to do further investigations (3, 5, 6, 9, 13). However, these small studies have indicated that the use of US could be helpful as much as plain radiography in diagnosis of metatarsal fractures. 

Based on above-mentioned points, the aim of this study was to determine the diagnostic value of ultrasound in diagnosis of metatarsal bone fracture in patients referring to the emergency department due to foot and ankle blunt trauma.

## Methods

***Study design and Setting***

This cross-sectional study was carried out on patients admitted to emergency department of Edalatian Hospital, Mashhad, Iran, from January to September 2016, following foot trauma. All patients underwent ultrasonography and radiography of foot and diagnostic accuracy of ultrasonography in detection of metatarsal bone fractures was measured. This study was approved by the ethics committee of Mashhad University of Medical Sciences, Iran (Code: 931645). In addition, all items expressed in the Declaration of Helsinki were taken into account. The patients filled the written consent form for enrollment in the study. Given that our study was an observational one, there was no threat to patients for therapeutic research efforts; however, the researchers supported all patients during the study in case of any complication.

***Participants***

All conscious cases over 16 years old with stable hemodynamics and clinical indication for foot radiographic examination were enrolled to the study. Lack of patient consent, penetrating trauma, open fracture or dislocation, and the presence of osteomyelitis or chronic skeletal problems were among the exclusion criteria.

***Data gathering***

Basic information, including age, gender and type of trauma (penetrating or blunt) was recorded for patients. Then, the emergency medicine specialist performed bedside ultrasound for all patients and ultrasonography findings regarding presence or absence of metatarsal bone fractures were recorded. Then, all patients underwent foot radiography (posterior-anterior and oblique views). The emergency medicine specialist, faculty member with board certificate, interpreted radiographic findings. In addition, a radiologist as a project colleague interpreted all radiographs. The radiographic findings were recorded as the presence or absence of fractures. The emergency medicine specialist was unaware of radiographic findings when doing the ultrasound and the radiologist was also blind to the sonographic findings. The emergency medicine resident was responsible for data gathering. In this study, the samples were selected by nonrandom purposive sampling method. 

All sonographies were performed according to Ankle Ultrasound Technical Guidelines of European Society of Musculoskeletal Radiology, using ultrasound device (manufactured by Honda - Japan) and 10 MHz linear probe. In addition, the bone structures that should have been evaluated by ultrasound were prepared in a checklist in advance for specialists to assess the items based on the checklist. The bone checklist included first to fifth metatarsals. The fracture diagnosis criteria in ultrasound were the presence of cortical disruption or stepping or axial deviation of the bone surface.

***Statistical Analysis***

Given the 60% prevalence of metatarsal bone fractures and 87.3% sensitivity reported in the study by Atilla in 2014 (13), as well as taking into account the type I error of 0.05, L=0.06, the sample size was calculated as 72. Considering a 20% dropout rate, the final sample size was 90 patients. 

All data obtained from the patients were entered into the computer and statistically analyzed using SPSS 17.0 for Windows (SSPS Inc., Chicago, IL, USA). Given the normal distribution of data, quantitative data were expressed as mean and standard deviation. The sensitivity, specificity, positive predictive value (PPV), negative predictive value (NPV), positive likelihood ratio (PLR), and negative likelihood ratio (NLR) of sonography in detection of metatarsal bone fractures were calculated with 95% confidence interval (CI) using the Medcalc version 16.1 software. P<0.05 was considered statistically significant.

## Results

***Baseline characteristics of participants***

The study was conducted on 102 patients with a mean age of 35.14±14.32 years (56.8% male). The trauma had happened during everyday life in 63 (61.8%) patients, due to accident in 27 (26.5%) patients, and during exercise in 12 (11.8%) patients. The most common signs of trauma in physical examination were pain and tenderness (100%), swelling (96.1%), ecchymosis (14.7%) and deformity (1.9%). The mentioned signs were frequently detected in fore-foot (> 70%). 

***Diagnostic measures***

The prevalence of metatarsal fracture was 30.3% (n=31) based on radiographic findings and 40.1% (n=41) according to sonographic results.

**Figure1:**
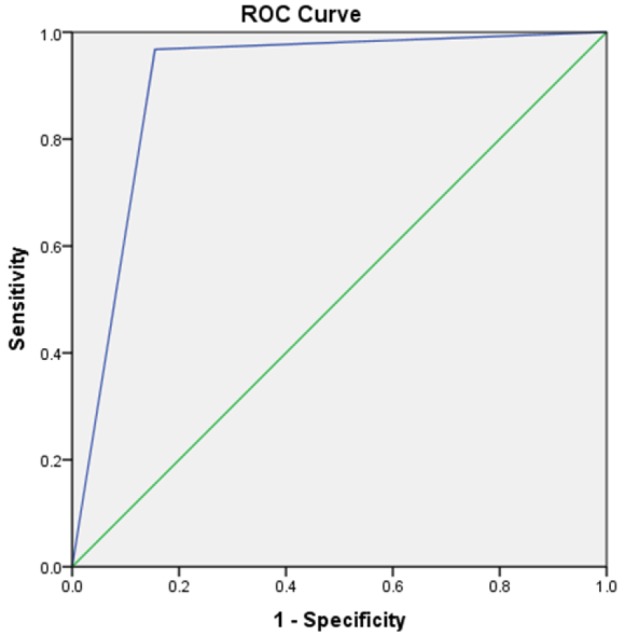
Area under the receiver operating characteristic (ROC) curve of ultrasonography in detection of metatarsal bone fracture

Overall, the ultrasound did not detect the fracture for one patient (medial side of the fifth metatarsal). In Addition; 11 (27%) suspected fractures on ultrasonography were not confirmed by radiography. The sensitivity, specificity, PPV, NPV, PLR, and NLR of ultrasonography in detection of metatarsal bone fracture were 96.7% (95% CI: 0.83-0.99), 84.5% (95% CI: 0.73-0.92), 73.1% (95% CI: 0.57-0.85), 98.3% (95% CI: 0.91-0.99), 6.25 (95%CI: 3.61-10.79), and 0.04 (95%CI: 0.01-0.26), respectively. The overall accuracy of ultrasonography based on area under the receiver operating characteristic (ROC) curve was 0.906 (95% CI: 0.844 – 0.969, figure 1).

## Discussion

The results showed that ultrasound has remarkable sensitivity and specificity compared to radiography and can be used in a shorter time than radiography in emergency situations for diagnosis of metatarsal bone fractures.

The use of ultrasound for diagnosis of fractures by emergency physicians is more important when the priority is deciding within the shortest possible time, especially when the radiography ward is overcrowded and there is the need to speed up the triage of patients to properly manage the emergency department. In addition, sometimes a doctor has high clinical suspicion to the existence of concurrent soft tissue damages in patients admitted to the emergency department. In these cases, due to higher power of ultrasound in diagnosis of soft tissue injuries compared to radiography, using ultrasound can be a priority in diagnosis.

In order to use ultrasound in diagnosis of fractures in the emergency department, knowing the diagnostic power of ultrasound for observing the fractures is important. This issue is something that very few studies have examined as many articles related to the use of ultrasound in diagnosis of metatarsal fractures are case reports (4-8, 11, 12).

Preliminary results of this study confirmed that ultrasound was a powerful tool in triage of patients referring to the emergency department with suspected metatarsal bone fracture; this result is consistent with two other studies in this regard. In a study by Ekinci et al. (14) sensitivity, specificity, positive predictive value and negative predictive value of ultrasound in diagnosis of metatarsal fractures were reported as 100%, 99.1%, 95.2% and 100%, respectively. In a study by Canagasabey et al. (2) sensitivity, specificity, positive predictive value and negative predictive value of ultrasound in diagnosis of fractures were found to be 90.9%, 90.9%, 52.5% and 98.9%, respectively. The sensitivity was over 90% and specificity was over 85% in both studies, similar to our research. High sensitivity and specificity are two important features in determining the power of a diagnostic tool; hence, ultrasound seems to be a powerful tool in diagnosis of fractures. PPV was reported as 73% and NPV was 100% with upward approximation. High negative predictive value of ultrasound may be the most important parameter confirming the diagnostic value of ultrasound; because if ultrasound is negative, the presence of fracture can be ruled out, leading to better triage of the patients. 

However, since PPV and NPV are related to incidence rate of the disease in the study population and fracture prevalence rates are different among various studies, likelihood ratio, which is not under influence of disease prevalence, is used for comparison. In the present study, PLR and NLR were reported as 6.25 and 0.04, respectively, indicating the high diagnostic value of ultrasonography. 

It seems that the high diagnostic value of ultrasound in diagnosis of metatarsal fractures can be attributed to the dynamics of ultrasonography and the ability of the operator to create images in different sections. It should be noted that the reason for slight differences between the results of the current and other studies regarding sensitivity, specificity and other parameters of diagnostic power could be due to differences in sample size, different quality of ultrasound devices used, experience and academic ability of ultrasound operators. 

Importantly, about 4% of patients in our study were pregnant, which highlights the importance of using x-ray-independent methods to diagnose fractures. Moreover, since the physician responsible for the ultrasound can press the probe when performing ultrasound examination, fractures with bone dynamic movements are more easily detected during ultrasound, and the pressure mentioned in previous studies will not be accompanied with unbearable pain for patients (3).

The gold standard for assessment of fracture in this study was radiographic findings interpreted by the radiologist. This criterion was chosen because plain radiography is performed in almost all patients with suspected fractured foot trauma. Selection of CT scan as the standard criterion would impose excessive and sometimes unnecessary radiation on the patients and MRI was associated with high and unnecessary time and cost.

## Conclusion:

According to the results of the present study, ultrasound has high sensitivity, high specificity, negative predictive value of about 100%, and favorable likelihood ratios in diagnosis of metatarsal fractures. Therefore, it seems that use of ultrasound can serve as a very good alternative to radiography in diagnosis of metatarsal fractures.
